# What is consciousness and what it is for. An introduction to extended information theory

**DOI:** 10.3389/fpsyg.2025.1627289

**Published:** 2025-09-03

**Authors:** Bruno Forti

**Affiliations:** Department of Mental Health, Azienda ULSS 1 Dolomiti, Belluno, Italy

**Keywords:** phenomenal consciousness, function, phenomenal analysis, hierarchy of spatial belongings, extended form, extended information theory

## Abstract

In this paper, I outline a new theory on consciousness, the Extended Information Theory. This theory jointly addresses issues related to the nature of consciousness and those related to its functional role. The phenomenal analysis of the simplest aspects of experience allows us to identify the structure of consciousness within consciousness itself. The simplest forms of experience are not found in sensations, but in forms of perception in which the qualitative aspects of consciousness necessarily have relational significance. Furthermore, this analysis leads us to hypothesize that the structure of an early visual experience is constituted by a Hierarchy of Spatial Belongings nested within each other. This structure makes it possible to identify a property of consciousness that is more fundamental than qualitative aspects. It can be identified in the fact that a conscious content, like an object, extends in a certain way into the space to which it belongs. Even when faced with an unfamiliar image, this allows us to know how its contents extend into the space to which they belong. The primary role of consciousness could thus be identified in knowing, in the immediacy of experience, the structural aspects of the physical world that surrounds us. From a functional point of view, it can be stated that consciousness handles Extended Information and differs from Non-Conscious systems that handle point-like information. It is in this characteristic, which enables it to overcome some of the limitations of computation, that the evolutionary meaning of consciousness may lie. The phenomenal analysis of early perception allows us to examine this process of knowledge and to propose a tentative hypothesis regarding its functioning. Finally, the paper discusses the difference between the EIT, which reflects the need to integrate information about the structure of the stimulus, and theories based on classical integration.

## 1 Introduction

The purpose of this paper is to identify the fundamental property and function of consciousness. I argue that they coexist in a single functional property. This property makes it possible to outline a new theory on consciousness, the Extended Information Theory (EIT). I will also present a tentative hypothesis of how consciousness performs this function and what its role is in the functioning of the conscious mind. I will focus almost exclusively on visual perception. This means that the EIT, as presented here, does not apply to all sensory modalities and forms of consciousness.

We think we know the properties of phenomenal experience (James, 1890; Tononi and Edelman, 1998; Zeman, 2001; Edelman, 2003; Searle, 2004). In most cases, experience is identified in qualia (Chalmers, 1995; Banks, 1996; Koch, 2004; Sturm, 2012; Grossberg, 2017; Tsytsarev, 2022). Consequently, we search for an explanation of how experience can occur in classical neuronal organization (Crick and Koch, 1998; Feinberg and Mallatt, 2013; Gallotto et al., 2017; Orpwood, 2017; Noel et al., 2019; Tyler, 2020; Vallortigara, 2021; Seth and Bayne, 2022) or outside of it, for example in magnetic fields (McFadden, 2020, 2023; Ward and Guevara, 2022; Jones and Hunt, 2023; Hunt et al., 2024; Strupp, 2024) and quantum physics (Hameroff and Penrose, 2014; Beshkar, 2020; Tuszynski, 2020; Zhi and Xiu, 2023). It is believed that progress can only come from this approach. There are probably two reasons for maintaining this stance. Firstly, qualia seem to represent consciousness both in their simplicity and in their specificity (Shoemaker, 1991; Kind, 2008; Loorits, 2014; Kauffman and Roli, 2022; Arakaki et al., 2023). Secondly, the hard problem seems to be precisely about qualia (Chalmers, 1995, 1996; Kanai and Tsuchiya, 2012; Frankish, 2012).

Instead, I believe that the fundamental property of consciousness has not been identified, and that progress in understanding consciousness is only possible through further investigation of experience: this is what I call Phenomenal Analysis. One of the difficulties in understanding consciousness lies in the belief—which I view as erroneous—that its simplest aspects are non-structural in nature (Loorits, 2014). Phenomenal Analysis, which I described in a previous paper (Forti, 2024a), can identify the structural nature of consciousness by analyzing the phenomenal and qualitative aspects of experience. This analysis primarily focuses on basic forms of experience, trying to prioritize the aspects that seem to belong to the fundamental framework of consciousness and might be involved in the formation of its structure and function.

The problem of how the brain generates experience is not only difficult, but also posed incorrectly. Identifying qualia as the starting point for a theory of consciousness has two limitations. Firstly, it is difficult to analyze these seemingly non-structural aspects in structural terms. Secondly, it is difficult to identify the functional role of consciousness in qualia.

The phenomenal analysis of the simplest aspects of experience allows us to identify the structure of consciousness within consciousness itself. Sensations such as the redness of red or the painfulness of pain are inseparable from the context of the experience to which they belong, making qualia appear as phenomenal artifacts. Hence, the simplest forms of experience are not found in sensations, but rather in forms of perception in which the qualitative aspects of consciousness necessarily have relational significance (Forti, 2024a). Furthermore, this analysis leads us to hypothesize that the structure of an early visual experience is constituted by a Hierarchy of Spatial Belongings (HSB) nested within each other (Forti, 2024b). Every spatial belonging is made up of a primary content and a primary space, which is not perceptible. In this sense, the structure of consciousness is counterintuitive because it is also made up of hidden parts.

Moreover, the problem of qualia should be closely associated with the—equally unresolved—problem of their functional role. Kanai and Tsuchiya (2012) state that “neuroscientists track how light impinging on the retina is transformed into electrical pulses, relayed through the visual thalamus to reach the visual cortex, and finally culminates in activity within speech-related areas causing us to say ‘red’. But how such experience as the redness of red emerges from the processing of sensory information is utterly mysterious.” This means that, at least with respect to these aspects, we already know the relations with a functional meaning. Even if we managed to solve the qualia problem, we would have explained something that adds very little to our understanding of mental functioning.

Why is it difficult to attribute a function to qualia? Based on common sense, we tend to attribute a functional meaning to feelings of cold, pain, or sweetness, as well as to the information we get from seeing the color green. However, it is very difficult to understand the extent to which these sensations provide an advantage over simply receiving the signal. At first glance, what makes them useless duplicates is the elementary, essentially non-structural nature of the simplest qualia. As such, they can easily be “replaced” by the corresponding reception of the stimulus. Again, placing the qualitative aspects of consciousness in a structural context like perception might make the task easier.

We still do not know the biological function of consciousness. Theories of consciousness proposed in recent decades “are concerned primarily with how consciousness arises, and only secondarily, if at all, with the biological function of consciousness” (Earl, 2014). Many authors do not attribute a function to consciousness (Velmans, 2002; Pockett, 2004; Rosenthal, 2008; Blackmore, 2016; Halligan and Oakley, 2021). However, from an evolutionary perspective, it is difficult to deny its adaptive role (Nichols and Grantham, 2000; Earl, 2014; Lacalli, 2024). Various functions have been attributed to consciousness (Edelman, 2003; Carruthers, 2004; Bayne, 2010;^133,75^; Ludwig, 2023). The functional role of information integration (Morsella, 2005; Tononi, 2008) will be analyzed below. Several scholars highlight the role of feelings and emotions in helping the individual to make decisions by weighing various behavioral options (Cabanac et al., 2009; Damasio and Carvalho, 2013; Damasio, 2021; Solms, 2021; Grinde, 2024). Consciousness is associated to the performance of complex tasks, particularly when these are novel, or require flexibility (Baars, 1997; Velmans, 2012; Earl, 2014; Lacalli, 2024). Mogi (2024) identifies “several cognitive domains potentially unique to consciousness, such as flexible attention modulation, robust handling of new contexts, choice and decision making, cognition reflecting a wide spectrum of sensory information in an integrated manner.”

It should be emphasized that many of the existing functional hypotheses do not take into account the phenomenal aspect (Block, 1995; Dehaene et al., 1998; Lamme, 2010; Feldman, 2013; Dehaene, 2014; Graziano and Webb, 2015; Brown et al., 2019; Yurchenko, 2022). According to Niikawa et al. (2022), there is no *a priori* reason to reject the possibility of there being nomological connections between phenomenal consciousness and cognitive functions. In my opinion, the function of consciousness should be closely related to the fundamental properties of experience. Otherwise, the risk is to identify a non-conscious function, dissociating the phenomenal aspect from the functional one (Chalmers, 1995; Solms, 2021; Seth and Bayne, 2022). Consequently, we should ask ourselves: what is the fundamental property of consciousness that can be an expression of its functions? This is not an easy question to answer (Velmans, 2012).

It is possible to identify three requirements for there to be a conscious function (Table 1). Firstly, if the function is closely related to the properties of experience, it becomes necessary to distinguish between processes and contents of experience (Velmans, 1991; Dehaene and Naccache, 2001; Umiltá, 2007; Korteling et al., 2021). Many believe that experience is nothing more than the result of a non-conscious process (Wegner, 2002; Robinson, 2007; Earl, 2014). Secondly, consciousness should have selected itself to solve problems that non-conscious brains had difficulty solving (Wiest, 2025). Such a hypothesis is difficult to make because, potentially, a non-conscious processor can cope with any problem in the—broadly speaking—cognitive sense. Thirdly, to have a function, consciousness must play a causal role on computational processes but cannot be caused by them (Forti, 2009). Even if a certain type of computation produced a phenomenal effect, the function would still be purely computational. The phenomenal component would merely be a side effect. A plausible solution is that it is a non-computational function, not originated by neuronal computational processes. Non-computability of consciousness would be supported by some evidence (Song, 2007; Hameroff and Penrose, 2014; Kak, 2024).

**Table 1:** The requirements for there to be a conscious function.

Three requirements for conscious function
1. The function is closely related to the properties of experience
2. Consciousness solves problems that non-conscious brain finds challenging
3. It is a non-computational function

These requirements seem to further complicate the hard problem. However, it is possible that addressing these two issues at the same time is simpler, or at least makes more sense. Phenomenal analysis makes it possible to identify the fundamental property of experience and to highlight its functional nature. As we shall see, the way consciousness functions can be derived from the phenomenal datum concerning *how* we know the world through conscious perception.

The problem is where to look. The aspects related to the quality of experience are undoubtedly one of the properties of consciousness and need to be explained. However, they are inseparable from the perceptual context of the experience to which they belong. The fundamental property of consciousness is to be found in perception and in its structure. Perception should also be understood in its counterintuitive aspects: the fundamental relationship is between content and space of belonging, its spatial belongings overlap widely, and its structure is composed of non-apparent parts (Forti, 2024b).

In this paper I argue that the fundamental property of consciousness is that its objectual contents extend into space. A conscious content, like an object, cannot but extend into the space to which it belongs. But we cannot simply say that a conscious content is extended. If an object that extends has a sufficient level of definition, it cannot help but extend in a certain way. Thus, it can be stated that the functional property of consciousness is that its objectual contents extend in a certain way into the space to which they belong. This allows us to know, at a more complex level, what the world around us is like.

It should be clarified that the term fundamental is primarily meant to refer to the fundamental aspects of consciousness, which I have identified in perception. The simplest forms of experience are not found in sensations, but rather in early perception (Forti, 2024a). Furthemore, perceptual aspects are present in many phenomenal experiences that are not strictly perceptual. Our experiences of mental imagery often feel perceptual, as if we are seeing, hearing, or touching things despite the absence of external stimuli. Symbolic thinking uses discrete tokens with references. Emotions usually overlap with an object - especially if we understand objects as something extended. In any case, in these experiences we continue to perceive, albeit in the background, our body and the world around us.

## 2 The cell of consciousness

Is there anything for consciousness that can be compared to what the cell is for a living organism? The cell is the building block of which a living organism is made, and it is the basis of its fundamental properties, such as duplication and differentiation. The cell of consciousness should be something that has the basic features of phenomenal experience, as well as functional features. Phenomenal qualities do not seem to be a suitable candidate, as they must necessarily belong to a perceptual context. On the other hand, what it is like (Nagel, 1974) is a vague and imprecise concept, presumably referring to a set of several closely intertwined components, such as more or less complex qualitative aspects, subjectivity and value connotations.

In order to find the simplest form of consciousness, we can progressively subtract aspects of our experience that can—at least temporarily—be set aside in elementary consciousness: for example, being aware of being conscious, the qualities of an object (Forti, 2024a), and the subject itself, as it is negligible in certain perceptual experiences (Tononi and Koch, 2008). But what cannot be eliminated in any way is the object and its relationship to its surroundings. As Merleau-Ponty (1945) states, “the perceptual ‘something’ is always in the middle of something else, it always forms part of a ‘field’.”

It is quite easy to identify the fundamental property of consciousness by starting with the object and its relationship to the surrounding space, although this property is so obvious that it goes unnoticed. The usual focus is on the fact that each component of a perceptual field has a quality as an object, background, detail, and so on. But apart from these differences, these components share a common property: *they are extended*. Even phenomenal space appears extended to us (Haun and Tononi, 2019). The extended nature of consciousness is more evident in objects. But it is also present in sensations (Husserl, 1913; Merleau-Ponty, 1945), all the more so if we keep in mind that they overlap with objects (Forti, 2024b). Symbols also have a pictorial and extended perceptual nature.

**Figure 1:**
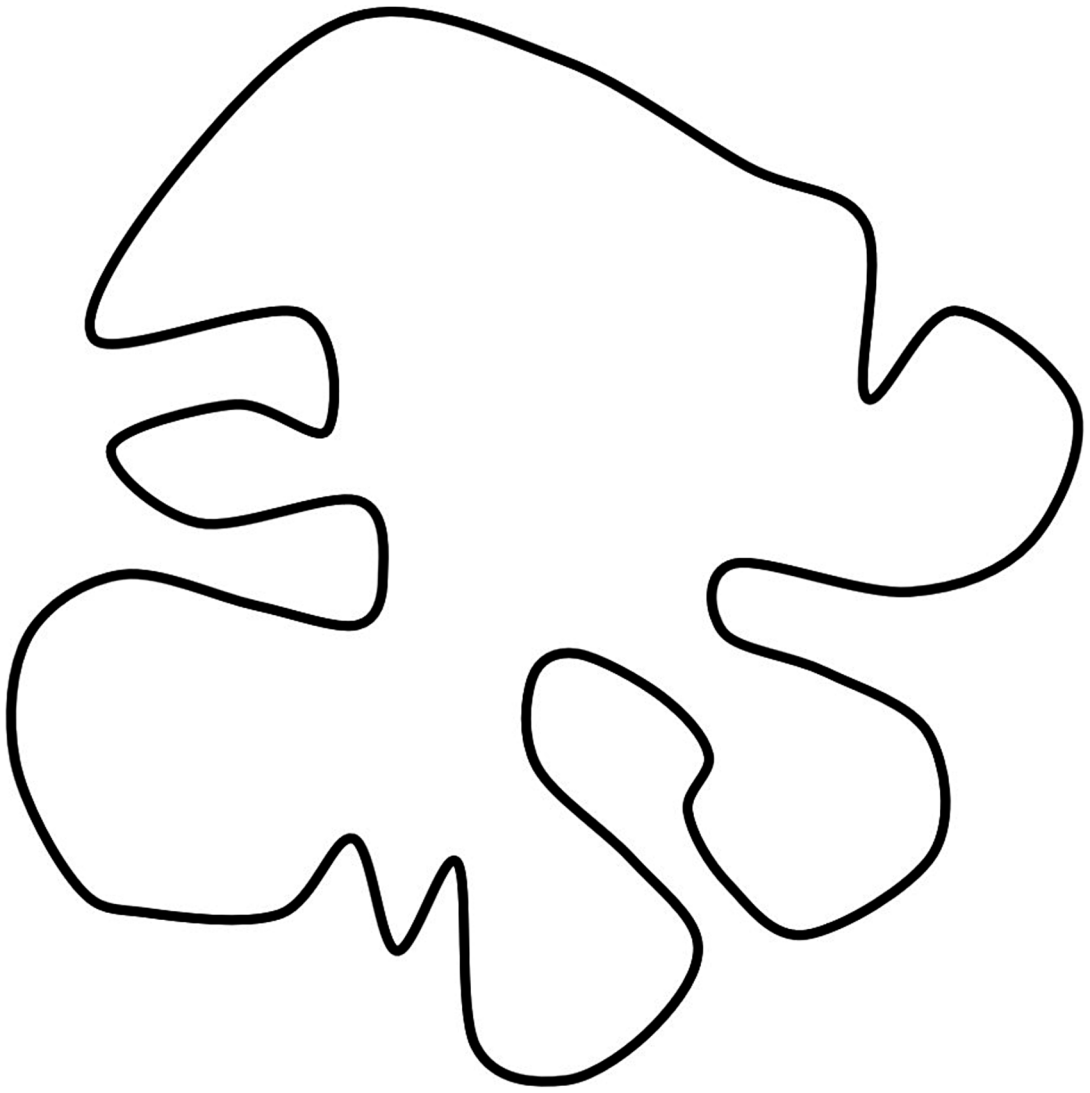
A shape on a sheet of paper. Seeing how the figure occupies the available space is independent of the complexity of the form and whether it is known.

The phenomenal evidence that an object is extended into space carries with it another evidence. If the object is sufficiently defined, identifying its extended nature also means identifying how it extends. To a certain degree of approximation, something similar to what happens in a cell of consciousness can be found in the perception of shape on a sheet of paper (Figure 1). It can be useful to ideally refer to an unknown form Kanizsa (1991). When we are visually exposed to such an image, we cannot help but see how the figure occupies the available space of the sheet, in other words how it extends into the space to which it belongs.

I use this terminology because, in this kind of situation, vision seems capable of gathering a wide range of information that can hardly be summarized with the usual terminology. Even at a very early stage of perception, regardless of existing knowledge, the perception of that figure seems to provide answers to questions such as: where is the figure located in the defined space? What are its dimensions relative to the space in which it is located? What shape does it have? How does it move, i.e., how does it change location, shape and size over time? Remarkably, this ability appears to be independent of the complexity of the form and whether it is known (Figure 1).

The property of a simple figure to extend in a certain way into the space to which it belongs is a specific property that incontrovertibly identifies what is conscious and differentiates it from what is not conscious. To use Nagel’s (1974) terminology, it can be stated that *an organism has conscious mental states if and only if the contents of these mental states extend in some way into the space of belonging*. At the same time, this property is more fundamental than a property such as what it is like.

Extending in a certain way is not only a property related to how objects appear. As it implies knowledge, it is a property that can be related to conscious function, hence to the handling of information (Lycan, 1996; Mangan, 1998; Earl, 2014). I assume that, in order to know how an object extends, consciousness must handle extended information. Extended information is characterized by the fact that its informational units are objects or entities that are spatially extended in some way and cannot be reduced to point-like entities. I call Extended Information Theory (EIT) the theory according to which consciousness is characterized by handling Extended Information.

I introduce the term Non-Conscious (NC) process to refer to a process that occurs in the absence of consciousness. To a certain degree of approximation, the acronym can also be interpreted as Neuro-Computational and include both artificial processes and processes carried out by the brain in the absence of consciousness (Forti, 2009). The argument that consciousness handles Extended Information acquires an important meaning if we note that the information processed by a NC system is necessarily point-like information. This is, again, a property that is so obvious that we are not aware of it. One reason is that we are convinced that it can only be so.

The fundamental difference between non-conscious and conscious processes is that the former handle Point-Like Information, while the latter handle Extended Information. If we accept this dichotomy, then all information-related systems should be distinguished based on whether they are extended or point-like, depending on whether they are conscious or NC systems, respectively. Consciousness is different from a NC system in that it is characterized by extended knowledge, perception, integration, images, form, and structure. I will elaborate on the meaning of this term later in this paper.

So, what is the function of consciousness? The one I have described relates to an elementary case: how a simple shape extends into the space to which it belongs. Although it is phenomenologically grounded, it is a simplified and unrealistic situation compared to normal conditions of stimulation. In a situation more complex than a simple shape, we have to take into account that numerous spatial belongings overlap and nest in each other (Forti, 2024b). For example, in a face the belonging of the eyes to the face overlaps with the belonging of the face to the surrounding space. Each individual eye belongs to the gestalt of the pair of eyes. So even two objects that we perceive as juxtaposed should be considered as both belonging to the space of the gestalt they form.

In addition, while limiting ourselves to vision here, we should take into account the overlapping of images from other senses. If we broaden the field to multi-layered visual organization and multimodal perception, it can be argued that *conscious knowledge derives from the way in which the contents of an image extend into the space they belong to and overlap with each other*. It includes not only form, location and size of a single figure, but also the overlapping of multiple contents in an image and the overlapping of multiple images. This high level of overlap is made possible by the phenomenally negative nature of primary spaces (Forti, 2024b).

What I have described above refers to the main belonging of a HSB. Therefore, it should be pointed out that the possibility of knowing how a content extends into space is not evenly distributed in the field. It is greatest in the focus and gradually fades in other areas of the field. However, the variable structure of the HSB causes the focused content to continuously change according to both the stimulus and the NC processes involved. This way, it is possible to progressively achieve integrated knowledge of the entire image.

## 3 What is a bat like?

The above makes it possible to propose a more intuitive, albeit rough, definition whereby *consciousness enables us to know what the world around us is like, what its structure is*.

If we come across a bat, consciousness can help answer the following question: what is a bat like? According to Nagel, the starting point for understanding consciousness is to ask ourselves what it is like to be a bat. From my perspective, however, the starting point is simpler: to ask ourselves what a bat is like. In fact, it is a type of knowledge that we do not usually take into consideration. For example, it is a knowledge other than knowing as truthfulness, i.e., that knowledge whereby, if I perceive an apple before me, then there must be an apple (Reid, 1764/1977, in Reeves and Dresp-Langley, 2017).

Three main types of knowledge are typically distinguished: know-how, know that, know somebody or something (Garud, 1997). Conscious knowledge seems to most closely resemble knowing somebody or something, which, on a surface level, can be likened to acquaintance. According to Russel (1912), “we have *acquaintance* with anything of which we are directly aware, without the intermediary of any process of inference or any knowledge of truths.” Conscious knowledge is extended knowledge. It is not only knowledge concerning how an object extends. Rather, it is knowledge itself that occurs in an extended form. This knowledge needs to be specified both with respect to what it refers (1–3) to and with respect to its characteristics (4–9) (Table 2).

1 By structure I mean the apparent structure of the world, i.e., the form, the morphology of the objects we find in it. This means, on the one hand, that it is not an internal or hidden structure. On the other hand, appearance is never limited to a single impression, but is always a structural fact. Even when a sensation is prevalent over other aspects we perceive, it is always embedded in a structural context.

**Table 2:** The extended conscious knowledge of the world with respect to what it refers to and with respect to its characteristics.

Extended conscious knowledge
*What does it refer to?*
1. It refers to the apparent structure of the world, i.e., to the form of objects
2. Form is the way in which an object extends into the space to which it belongs
3. The world is not just the distal stimulus
*What are its characteristics?*
4. It is a current knowledge
5. It has a spatial structure
6. it is knowledge by images
7. it is private knowledge
8. It is an integrated knowledge
9. It can be distinguished into primary and secondary perception

The apparent structure of the world is less trivial than one might think. The morphology of real objects is more complex than the objects that comprise the scientific view of the world, which can often be reduced to points or simple formulas. On the other hand, knowledge of the apparent structure of the world has significant adaptive value. It enables us to move and act in the world, to establish relationships—including through our own bodies—with the objects around us. Moreover, it is changeable. The pre-Socratic philosophers, starting with Thales, noted that appearances change, and began to ask what the thing that changes “really” is (Wikipedia Contributors, 2025). The distinction between apparent, changeable form and what should be the “actual”—real or ideal—form has persisted to the present day (Witzel and Gegenfurtner, 2018; Calì, 2020). With the exception of phenomenology (Husserl, 1,013), this distinction has led us to overlook both the importance of appearance in its changing nature and the difficulty of acquiring knowledge of it.

2 In the sense in which the term will be used here, form is the way in which an object extends into the space to which it belongs. As we have seen, extending in a certain way should be understood more broadly, i.e., in reference to the image, where not only form in the strict sense, but also the location, size and movement of the object matter. In an even broader view, form includes overlapping, which is the only possible relationship between contents—relating to the same object—that extend in a certain way into the space within an image. I call Extended Form (EF) the way in which the contents of an image extend into the space to which they belong and overlap with each other.

In this paper, the EF refers to the vision of an object, because exploring vision makes it possible to more clearly express concepts related to knowing *how* an object extends into space. In fact, from the earliest evolutionary stages of consciousness, what is known is likely not only a form per se as an expression of pure observation. It is also something that concerns non-preordained relationships between stimulus and organism response, which involve extended regions and extended interfaces between these regions. This may occur, for example, in handling an object, placing it in a container or moving through a rough environment (Billard and Kragic, 2019; Jacquey et al., 2019; Eikelboom et al., 2020). This is sensorimotor knowledge, which would occur in animals with consciousness, making it possible both to deal with certain unpredictable situations and to display flexible behavior (Earl, 2014).

3 By world I do not mean only the external world as it presents itself to us through current stimulation, i.e., distal stimulus. In addition to knowing the external world, consciousness also enables us to know the remembered, imagined, or dreamed world. This may seem counterintuitive, as it would not seem necessary to know a remembered object. However, it should be kept in mind that mental imagery recall involves remodeling memory. One could say that consciousness serves to know any image that accesses it. However, it is legitimate to retain the definition that consciousness serves to know what the world around us is like, because it refers to the fundamental function of consciousness. The imagined world, which we can model, is also part of the knowable world and must possess the fundamental characteristic of being formed by contents extended into space. We could not imagine or create an object without knowing what it is like.
4 In terms of its characteristics, conscious knowledge is current knowledge. It is only what we perceive, and therefore know, in conjunction with experience, even if it is influenced by what we already know. “Current” means that knowledge is both almost instantaneous and transient, fleeting.

Its existence is limited to conscious experience. Except for its brief retention in working memory (Baars and Franklin, 2003; Samaha, 2015), knowledge disappears as conscious knowledge when the corresponding conscious state ceases. Consciousness has no memory; it does not store the knowledge it acquires. Of course, conscious knowledge can be stored at the non-conscious level (Velmans, 2012) and later re-access consciousness. But each subsequent access of an image can only result in further knowledge, with a modification of knowledge itself.

At the same time, knowledge of how the figure extends seems almost instantaneous. It is acquired, without the need for learning, in the immediate interaction with the surrounding world. It provides knowledge about the current state of the surrounding world that is immediately available for action. The notion of access consciousness and availability for action (Block, 1995) should therefore be understood with reference to the property of consciousness to be immediate knowledge. We are aware that contents access consciousness, but not that this entails knowledge.

5 Conscious knowledge is characterized by a spatial structure. Acquaintance is typically taken to be simple and thus indefinable (Fumerton, 1995). In my view, conscious knowledge is instead—from the very foundation—structural. But defining conscious knowledge as structural is not enough. We have seen that knowledge concerns the apparent structure of the world. As such, it is simultaneous knowledge, made possible by a spatial structure. We do not have the experience of knowing the form of an object one piece at a time. Consciousness has a primarily spatial structure, based on simultaneous acquisition of information. In this, it differs from the structure of a computational system, which is primarily temporal, in that it is based on sequential operations. This does not mean that consciousness and a computational system cannot enable temporal and spatial knowledge of the world, respectively. The former is based on short-term memory, while the latter is based on the speed of its elementary operations.
6 A crucial aspect of extended knowledge is that it is knowledge by images. Forming a conscious image means knowing how an object extends into space. It should be pointed out that conscious images differ from, for example, retinal images or pictorial mental representations. I call these images NC images: they are such in that they retain certain topological relations present in the distal stimulus, but they do not in any way guarantee the occurrence of the experience, nor do they have any knowledge by images. The NC images present in our brains can represent the world pictorially and can be used for recognition, but NC knowledge is symbolic.

We tend to confuse a conscious image with a NC image. It is difficult to get an idea of what a pictorial representation actually is. In fact, if we see it or just imagine it, a process of knowledge is triggered whereby it is “transformed” into a conscious image. We are convinced that pictorial mental representations, even if unaccompanied by experience, represent knowledge of the world. At the same time, we think that our consciousness must be something more than just a consciously perceived image and that it is this something more that makes the difference. For example, we think that consciousness is the subjective perception of an image with the creation of a sense of self in the act of knowing (Damasio and Meyer, 2009), or the awareness of seeing something (Rosenthal, 1997; Brown et al., 2019), or the formation of a 3D image (Jerath et al., 2015), or our reaction to the perceived image—in other words what it is like to see an image (Chalmers, 1995)—or the attribution of meaning to what we see (Micher and Lamy, 2023).

However, conscious images are nothing more than the knowledge of the NC images. While a NC image has some structural correspondence with the stimulus, a conscious image is the knowledge of the EF of the former. In this difference lies the essence of consciousness, of its fundamental property and function. In this sense, what makes the difference is not the ability to generate representations (Kanai et al., 2019), but the ability to know their pictorial structure.

7 Extended knowledge is private. The definition of EF may seem different from form as understood in the common sense of the term. However, the way an object extends into space *corresponds to the form as we actually perceive it*. We do not define form in this way because our perception of a form is private knowledge. The idea of private, non-transmissible knowledge like acquaintance, which usually refers to qualities (McGinn, 2004), can also be applied to the EF. Although we *know* this, we are not able to specify *which way* an object extends into space, except to a limited extent (Block, 2011). We do not do so because of the limitations associated with how we share our private knowledge. While our conscious, private knowledge is extended, our transferable, shared knowledge is point-like.

If perceptually we determine what a polygon is like on the basis of the way it extends, of its occupying space, cognitively we define it on the basis of the straight segments that delimit it. We define a line as a succession of points. A figure itself is “thought of” as a continuous set of points. These definitions do not explain what a triangle actually is like: for example, they do not explain why a triangle appears “pointier” than a hexagon.

We know what a concavity is even without knowing its geometric definition, i.e., if a shape is concave, there is some place on it where a line drawn between two points in the shape will go outside the shape. Such a definition, albeit correct, seems unnatural to us compared to our perceptual experience. Of course, the gap between the two forms of knowledge is particularly evident in the case of complex, unknown forms (Kanizsa, 1991; Block, 2011) (Figure 1). The EF is essentially indefinable, and the definition I have given, namely that an object extends *in some way*, reflects this difficulty.

8 It is an integrated and unified knowledge. It is a whole made up of interdependent parts. I will address this aspect in the next sections. Again, it is an extended integration, different from what is commonly understood.
9 One problem with the different forms of knowledge is distinguishing the conscious component from the NC component. In this paper I limit myself to visual perception and I make a distinction which, in my view, is essential to understand conscious knowledge: the distinction between primary perception and secondary perception. It is based on Kanizsa’s (1979, 1980, 1991) distinction between primary process and secondary process (Luccio, 2003). The first one corresponds to the earliest form of visual experience, related to perceptual organization (Lamme, 2020). According to Kanizsa (1979), “the gestaltists have concerned themselves with the primary process. They have proposed a field model in which, by means of the dynamic self-distribution of the effects of the sensory input, phenomenological units or objects are generated with all their properties of color, size, shape, three-dimensionality, movement, and expressiveness.”

In my opinion, this description is the closest to a description of conscious processes, although this was not Kanizsa’s intent. Interestingly, the primary process is something fundamentally different from the processes which he calls thought processes—and which today we might equate with cognitive processing, not fundamentally different from NC processes. In perception, thought processes represent what he calls the secondary process. They involve recognition, semantic interpretation, or other higher cognitive processing of visual information.

However, even though Kanizsa’s description encompasses knowledge of objects, Gestalt theory addresses only perceptual organization. In this sense, my conception differs somewhat from the Gestalt approach, so I will use the terms “primary perception” and “secondary perception.” Primary perception is not merely perceptual organization. It is a knowledge of the images which access consciousness. As described above, primary perception ideally refers to an unknown form and it allows conscious function to occur in a sufficiently isolated form, without significant intervention of NC processes. In addition, these processes are not limited to primary perception, and secondary perception is not just recognition. As we shall see, in secondary perception the role of conscious processes remains knowledge, which, through interaction with NC processes, mainly addresses new aspects. This is how we come to know how the world deviates from our predictions.

## 4 Consciousness is an extended reference system

Consciousness enables extended, immediate, simultaneous and unified knowledge of the EF of the images that access it. This knowledge derives from the property of experience whereby its contents extend in a certain way into the space to which they belong. It is the fundamental property of consciousness, primary to other properties commonly attributed to it (Gomez, 2025). As such, it must be accepted as phenomenal evidence.

How does consciousness enable one to know what the world is like? Within the limits of this paper, it is only possible to make a tentative hypothesis. My proposal is that consciousness is a self-organizing extended reference system.

To gain immediate knowledge of non-predictable aspects of the world, a NC system can only use modalities that can be ascribed to a classical reference system. Of course, it can try to learn, but without gaining immediate knowledge. A classical reference system is based on a relationship between points located in a space. One point is the one we want to measure and the other two points are the origin and the unit of measurement placed along the axis starting from the origin. It is a point-like reference system. Through measurement, knowledge comes from matching the variable, i.e., the point we want to measure, with the multiples or submultiples of the unit of measurement, which is what I call the reference point.

A conscious reference system does not derive from relationships between points, or from relationships between objects that are comparable to points. A conscious reference system is based on the relationship between object and surrounding space. The object is extended and the space may be 1, 2 or 3D, but the system is “designed” for at least 2D spaces. It not only establishes the position of a point or set of points. It is able to detect—simultaneously and immediately—the position, size, shape and motion of an object, i.e., to know how an object extends into the space to which it belongs and how it changes its extension over time. For example, it can identify the motion of a bird taking flight, defining it by its trajectory, shape and size simultaneously.

There is no point that is the origin of the conscious reference system. The definition of the object occurs within a space. It is easier to think of this space as a frame in which the object is contained, i.e., as a well-defined space whose boundaries are sufficiently “close” to the object. At the same time, there is no unit of measurement or point of reference, because it is not a point, but rather an extended region within this space. We can call this an extension of reference. There is no correspondence with a point through measurement, but rather a deviation—or lack thereof—of an object from the extension of reference. Deviation can only concern the object and the extension of reference, in the sense of extended entities, in their entirety. In the sense used here, it may include a difference in shape, size or position. An important point is that the system is not measurable, because a measurement involves reducing what we want to measure to a point. An extended figure, as we consciously perceive it, cannot be reduced to a point.

In complex visual situations, such as those typically found in the stimulus field, there is not a single system, but rather a hierarchy of extended reference systems, corresponding to the HSB. Consciousness is unable to know the field in its entirety due to the overlapping reference systems. It is the one that prevails perceptually over the others that is known. However, since the different systems are nested in each other, this knowledge is not isolated, but integrated into a whole that can be explored progressively.

My argument is that the extension of reference corresponds to a condition of equilibrium in the relations between an object and the space to which it belongs. Knowledge of how an object extends into the space to which it belongs seems to depend on the degree to which it deviates—or does not deviate—from a condition of equilibrium in its relations with that space.

The hypothesis of an extended reference system can be derived from the phenomenal datum, from *how* we know the world through conscious perception. I will analyze the evidence showing that an extension of reference corresponds to a condition of equilibrium in the relations with the surrounding space in primary perception, in which consciousness ideally acts without the influence of NC processes. The case of the perception of a shape on a sheet of paper makes it possible to introduce the notion of a condition of equilibrium. When the space of belonging is well-defined and its contours are in relatively close contact with the object, the extension of reference coincides with the object being in a condition of equilibrium with it. Equilibrium means having the same shape, an extension half the size of the image, and being placed at the center of the image. Deviation can affect the figure in its entirety or parts of it. In the latter case, the form and relationships between the parts can be identified by observing how they deviate from the position of equilibrium.

The existence of a condition of equilibrium is phenomenally grounded because, when the figure as a whole deviates from an extension that is half the size of the image, we see it as large or small. We form our initial impression of an object’s size before making any comparisons with other objects based on the limits of the space in which the object is located. Similarly, we perceive an object as off-center (Arnheim, 1974) without the need to make measurements. The perception of shape also seems to depend on the contours of the figure, such as a protrusion or indentation that is not present in the contours of the image. If we see a square inside a circle-shaped image, we will tend to see the protrusion of the corners; if we see a circle inside a square-shaped image, we will tend to see the corners rounded off.

What happens when the influence of the limits of the external space is irrelevant, such as when its contours are distant and blurred? We can think of the condition of equilibrium as coinciding with something that has a uniform extension in all its parts, and thus with something that has no parts. Such a figure is nothing other than a circle or sphere. If we imagine a shapeless object, we usually attribute a roughly spherical shape to it. It should be kept in mind that the sphere is the simplest shape to define, that a point is the smallest perceivable sphere, and that we attribute a spherical shape to the simple components of matter, such as electrons. One could argue that, in primary perception, the shape of a figure in an undefined space is determined by the presence or absence of deviations from a uniform extension. This extension would have the same area as the figure and would roughly correspond to a circle extending around the center of gravity of the figure. Of course, a condition of equilibrium is not achieved solely through a circle. An internal articulation of the image may be compatible with a condition of equilibrium, even if to a lesser degree. Examples include multiple identical objects forming a gestalt, or an object composed of several identical parts, such as a hexagon or a stylized flower.

In perception we can identify objects or features of objects that might be called primitives of perception: for example, an oblong, crooked, wedge-shaped object; a protrusion or an indentation; an asymmetric or irregular shape. They are primitives of perception in that the simplest way to describe them consists of simple deviations from a uniform mode of extension. This is immediate knowledge that is not derived from prior knowledge. Nor do these primitives derive from calculations on entities made up of a set of points. We can perform these calculations a posteriori to check the accuracy of our perceptions. However, we would find it difficult to organize them coherently in the absence of conscious “guidance.” We do not need pre-existing knowledge or formal calculation to know what an oblong figure or protrusion is.

It is likely that a condition of equilibrium—which in an undefined space is achieved to a maximum extent in the sphere or a circle—is comparable to the Gestalt idea of good form or Prägnanz (Wertheimer, 1923; Kanizsa, 1980). The classical concept of Prägnanz has often been criticized and defined in different ways (Guberman, 2017; Luccio, 2019; Van Geert and Wagemans, 2023). However, if we take into account that form is an expression of the interaction between an object and the surrounding space, good form can mean equilibrium between these regions; consequently, a circle is an expression of good form (Köhler, 1922). Let us keep in mind that even a “zero” deviation from a condition of equilibrium represents a piece of information. Indeed, if the situation is not predictable, we tend to prefer a condition of equilibrium such as a circle, a right angle or a symmetrical object (Kanizsa, 1991).

It should be clarified that the contents of this section refer exclusively to the conscious reference system. As a rule, consciousness operates in cooperation with NC processes, which rely on classical reference systems based on spatial relationships between points. In an egocentric reference frame, the locations of objects are coded in relation to the observer (Moraresku and Vlcek, 2020). These systems can influence conscious perception by imposing constraints, such as left–right or up-down orientations, for instance when an object is expected to rest on the ground. The previously discussed examples of conditions of equilibrium do not take such constraints into account. However, when these constraints are considered, the amorphous block from which a statue is carved can represent a condition of equilibrium.

### 4.1 Explanatory hypotheses

In a previous paper (Forti, 2024b), I argued that qualities related to perceptual organization result from an interaction between primary content and primary space and from their overlapping in the HSB. The interactions concerning the main spatial belonging lead to the formation of object and background. It is possible to attribute the nature of forces to these interactions, which would also underlie conscious knowledge. They would act both within the field of the conscious image and toward the NC regions. In the former case they would produce knowledge, in the latter they would enable the transmission of knowledge to the NC system.

Above I suggested that conscious knowledge consists in the degree of deviation of the object from an extension of reference that represents a condition of equilibrium. This knowledge is likely made possible by the tension caused by the deviation itself and the simultaneous activation of forces in the field that work to reduce or eliminate it. We would expect the forces involved to reduce this deviation, as happens in physical systems. However, the conscious forces would be too weak to reduce the deviation by altering the relations between the parts, thereby producing changes in the contents of the conscious field. On the other hand, they would produce knowledge, which is the only obvious effect of the action of the conscious forces within the field and is observable only “from within.” This hypothesis is consistent with the presumed immateriality and causal ineffectiveness of consciousness.

**Figure 2:**
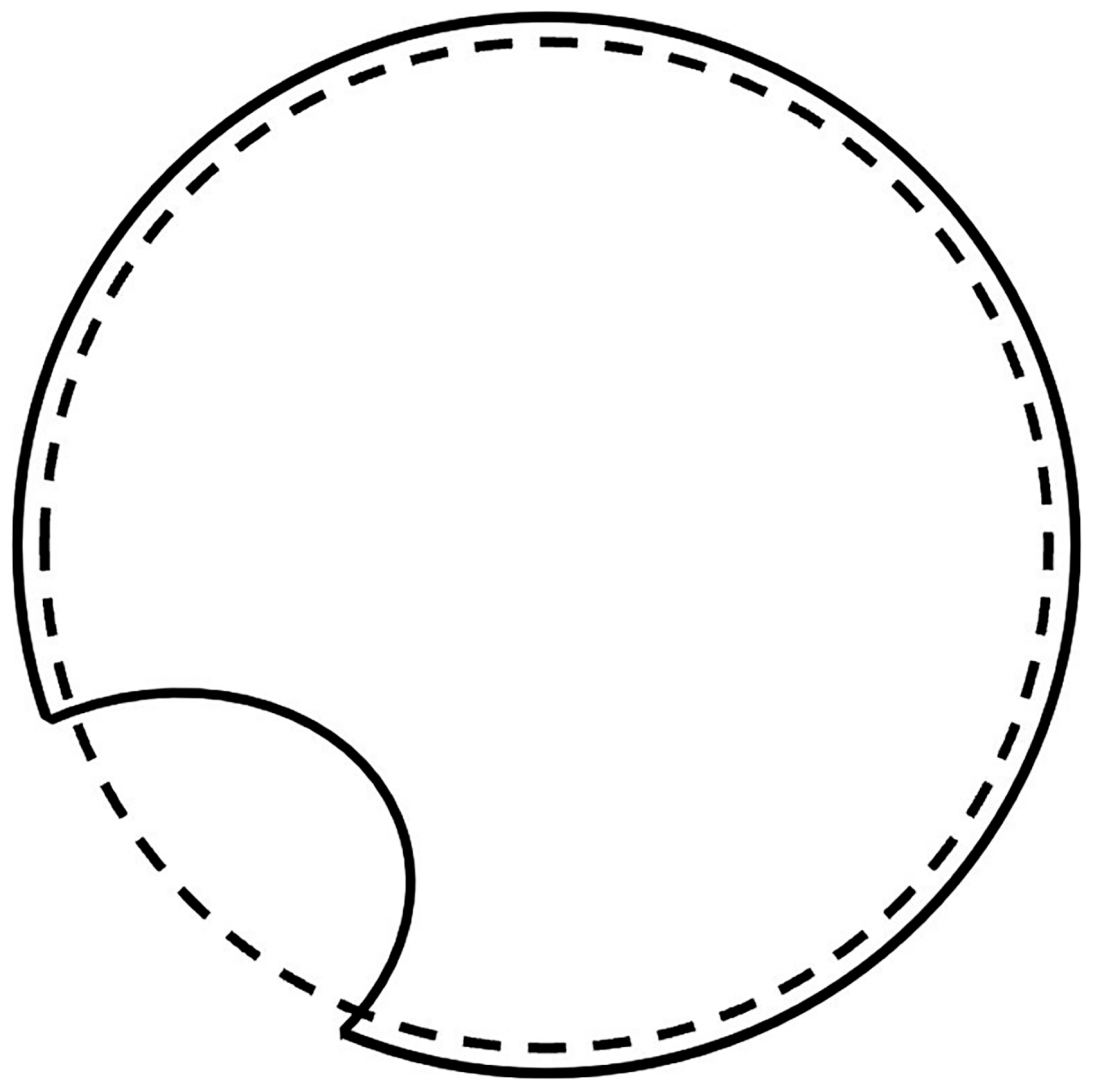
A circle with an indentation, representing the stimulus image, drawn with a continuous line, overlapped by the weak image, which represents the internally generated equilibrium condition, drawn with a dashed line.

One possible explanation of how the action of forces is translated at the phenomenal level is that an internal image forms and overlaps with the image corresponding to the stimulation. Once activated by the stimulus, consciousness would form a kind of *weak image* that represents the condition of equilibrium by which it provides information about whether or not the stimulus image deviates from it. Phenomenally, this weak image is nothing more than what makes us see an object as large or off-center in a box or makes us see an indentation in a circle when it overlaps with the stimulus (Figure 2). In contrast, we are impressed by a circle or regular figure because the extended form of the stimulus and the extension due to the condition of equilibrium are reinforced by converging on the same line of force. Thus, the phenomenal effect is greater. If the forces involved cannot change the relationships in the field, they affect NC activity. This represents the causal effect of consciousness on the NC system. This effect consists in the transmission of knowledge acquired by consciousness.

In the case where an object belongs to a space well defined by neighboring contours, perception is influenced by the size, position and shape of the object relative to the surrounding space. Contrary to what might be expected, the object in equilibrium does not seem to prevail perceptually. In fact, the influence exerted by the frame also depends on a tendency toward equilibrium. However, it involves two equally shaped regions that tend to be perceived together (Kanizsa, 1980). On the one hand, the condition with two equal shapes tends to prevail; on the other hand, it tends to make one see not the object located internally, but a gestalt formed by the two contours. Therefore, an object of a different shape than that of the frame is better seen as an autonomous object, even if it tends toward the unified perception of object and frame as a single gestalt.

### 4.2 Secondary perception

Primary perception is a somewhat ideal condition that allows us to observe consciousness in an isolated form. In the vast majority of cases, we know something about the world around us and experience secondary perception. What happens in these cases? First of all, conscious knowledge seems to be aimed at novelty, at what is not known by the system. When we perceive a known object, we tend to focus on what is changing and overlook the parts that confirm our expectations. However, secondary perception implies a set of relationships involving the object as a whole. The extended interface between new and unmodified part is also part of novelty. We see how the object changes more than we see the changed part itself. Knowing how the shape of an object changes is not just a matter of isolating the changed part and placing it in a “linear” relationship with the object.

Novelty may be total or, more often, partial. On the one hand, having some knowledge of the world—and thus activating expected images—is the norm in adult individuals. On the other hand, there is hardly any correspondence with the entire field. Since conscious processes involve multiple images overlapping in their wholeness, the presence of other elements of the perceptual field or a surfacing memory is sufficient to generate a deviation. Thus, consciousness almost invariably lies in the range between unknown and completely known.

One hypothesis that is compatible with the phenomenal datum is that there is first of all an interaction—mediated by NC processes—between stimulus image and known image. If the correspondence between known image and stimulus image is total, the NC system prevents access to consciousness. If the correspondence is partial, it decreases the salience of the unmodified parts, which tend to be placed in the background. In contrast, the new parts turn out to be salient. At the same time, conscious forces tend to convey the acquired knowledge of the new parts and of the changes in the relationship between known and new parts.

The acquisition of knowledge related to the stimulus image can cause conscious action to stop. In the case of progressive object knowledge, the known parts are placed in the background and salience gradually shifts to other aspects. Therefore, there is a kind of feedback between conscious and NC processes. Conscious forces tend to convey new knowledge, while NC processes tend to inhibit conscious activity when the image is known in part or in full.

## 5 Conscious knowledge and integration

All the above highlights the integrated nature of conscious knowledge. It could be said that, in world knowledge, integration arises from the need to know its apparent structure. Obtaining knowledge of the EF of an object means integrating, in the immediate term and simultaneously, all the information about how it occupies the space to which it belongs into a unitary whole.

The question of the unity of consciousness dates back to philosophers such as Descartes and Kant (Husserl, 1913; Searle, 1992; Nichols and Grantham, 2000) and has been addressed from multiple perspectives (Tononi, 2004, 2008; Bayne, 2010; Wagemans et al., 2012; Feldman, 2013; Chella and Manzotti, 2016; Amoroso and Rauscher, 2018; Hill, 2018; Masrour, 2020; Brogaard et al., 2021; Mason, 2021; Wiese, 2022; Hayashi and Sato, 2024). According to Morsella (2005), there is an “integration consensus” that consciousness functions to integrate neural activities and information-processing structures that would otherwise be independent.

Although they are very different from each other, three approaches take the phenomenal interdependence of the components of the perceptual field as their starting point. These approaches are (1) Consciousness-related binding, (2) the Integrated Information Theory and (3) Gestalt theory. First of all, unity has to do with the problem of binding, i.e., the connection between multimodal and submodal perceptions (Revonsuo, 1999). According to the Integrated Information Theory (Tononi and Koch, 2015), consciousness is unified, as each experience cannot be reduced to non-interdependent subsets of phenomenal distinctions. Gestalt theory emphasizes the mutual belonging between part and whole (Wagemans, 2012; Murgia et al., 2016).

What is overlooked is the form of the object. Binding does not address the relationship between the various components of the form of an object. Tononi and Koch (2015) ignore the most challenging structural aspect of Mach’s painting, namely the form of the observer. While Gestalt psychology claims to be a psychology of form, it limits itself to perceptual organization. The problem of the object form is only partially addressed, such as in symmetry (Wertheimer, 1923). Although Kanizsa’s (1979, 1991) description alludes to the knowledge of objects, Gestalt theory does not address the interdependence between the parts of an object as a constituent element of its knowledge.

In my opinion, these approaches address the problem of phenomenal unity, but come to a halt when confronted with form. Being an Extended Form, form is a watershed between shared, point-like knowledge and private, extended knowledge. Form in its internal relations is taken into account only in the simplest cases, for example with respect to the assembly or rotation of pre-formed components (Shepard and Metzler, 1971; Biederman, 1987; Xue et al., 2017). In more complex and unpredictable situations—which are the norm in the world around us—it becomes very difficult to describe it (Kanizsa, 1991; Block, 2011). Therefore, this is a limitation of our shared knowledge, which is based on relationships between points.

**Figure 3:**
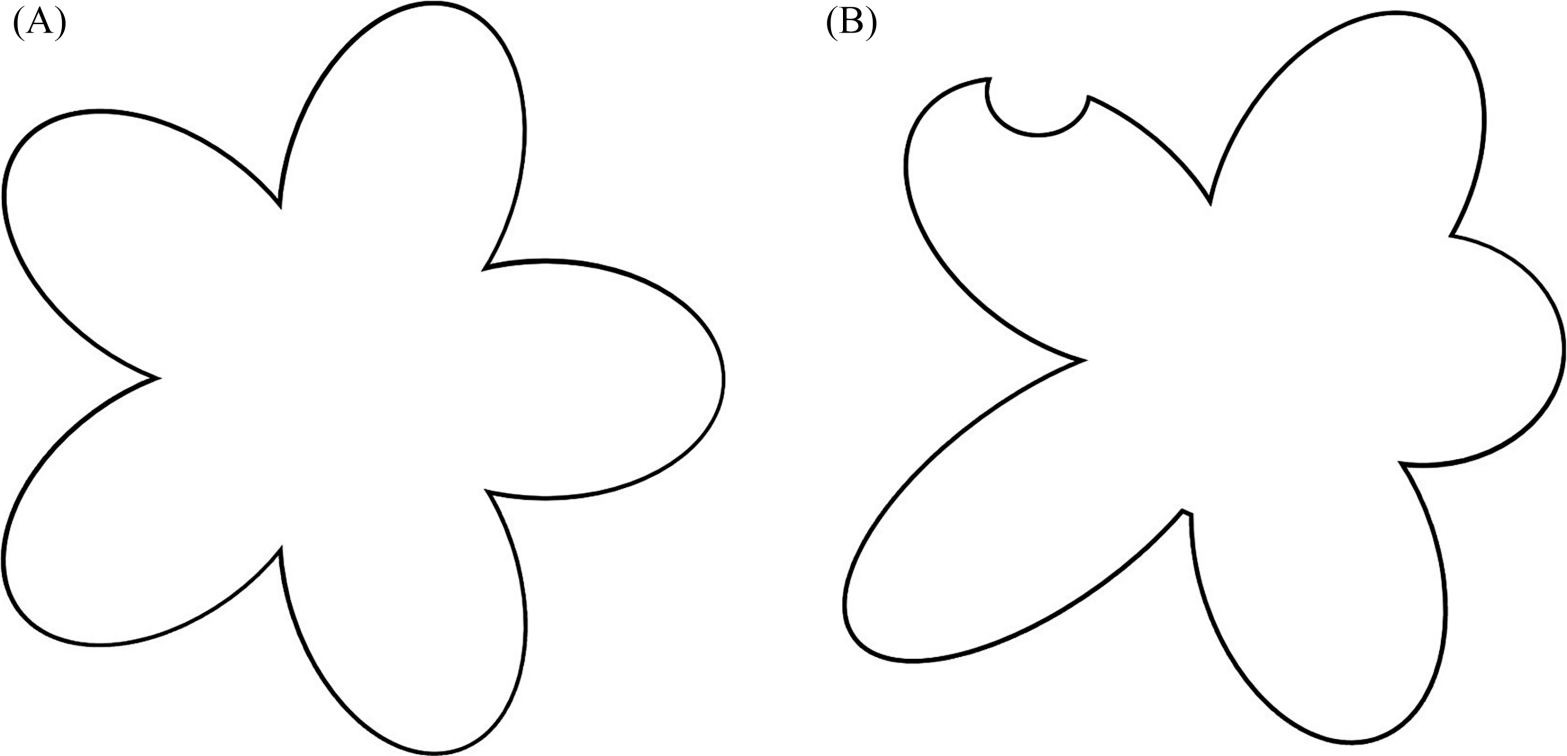
**(A)** A symmetric stylized flower **(B)** An asymmetric stylized flower.

A NC cognitive system is designed to cope with a world made up of points located in a space, not a world made up of objects extended into a space. Of course, nothing precludes it from treating the latter as a collection of points in relation to each other. However, a point-like integration can hardly handle complex and unpredictable structures in the immediate term when they involve objects that extend into the space to which they belong. In the case of the stylized flower shown in Figure 3A, a point-like integration establishes a relationship between the petals and, earlier, between the points that make up the contour lines. Of course, point-like integration can establish a relationship between the petals and the central body. However, the latter is simply one of the parts that is such when it can be distinguished from the others.

Instead, it seems that, in the perception of the form of an object, the parts are related not only to other parts, but also to something else that is not necessarily present in the stimulus. This something else could be identified in the extended region in equilibrium with the surrounding space—which I called the weak image. The relationship among the parts is secondary to this relationship. Thus, the region of reference is the pivotal element of both knowledge and conscious integration. The simultaneous interdependence among the parts of the object, which can be highlighted in the perception of form, depends primarily on this region.

The part is what deviates from a condition of equilibrium in a form. Some parts will simply deviate from a condition of equilibrium, such as a protrusion. Other parts will deviate from that condition but be in equilibrium with each other, like the sides of a regular polygon or the petals of a stylized flower, as shown in Figure 3A. Still other parts will deviate differently from a condition of equilibrium and some parts will deviate from the deviation, as shown in Figure 3B. A protruding part is only such in relation to all regions that are not protruding, to other equal protrusions and to different protrusions. Conversely, seeing a circle means seeing the whole contour equidistant from the center without making calculations. It is for all intents and purposes information that we have. This is how the deviation—or lack of deviation—involves the object as a whole.

While NC knowledge is not necessarily integrated and must be somehow supervised, conscious integration seems to depend on the nature of the processes at work. In consciousness, all the components of the perceptual field are phenomenally interdependent. This global, integrated nature of experience seems to depend on the tendency of the field to reach a state of equilibrium. It is the same that, as we have seen, underlies its knowledge, which is therefore integrated.

In the absence of consciousness, a non-conscious system can hardly immediately integrate a set of information concerning the object as a whole. But even in the presence of consciousness, transmission is not necessarily total. On the one hand, it is useful to know what the object is like in its entirety. On the other hand, it is sufficient to use that knowledge, for example, to know how to grab it. This view resembles the overflow argument (Block, 2011; Overgaard, 2018; Fu et al., 2021), according to which “perceptual consciousness is richer (i.e., has a higher capacity) than cognitive access: when observing a complex scene we are conscious of more than we can report or think about” (Block, 2011).

We thus have rich conscious knowledge and poor NC knowledge. Conscious knowledge—occurring in extended, simultaneous and unitary form—is rich, i.e., detailed, seamless in relation to the extension of the object and integrated in relation to the whole of it. NC knowledge is schematic, discontinuous, and made up of a set of point detections that are not necessarily integrated. The richness of conscious knowledge provides unitary guidance to the individual components of NC point-like knowledge, which would otherwise struggle to integrate with the wholeness of the object and image.

## 6 Discussion

The primary goal of this paper is to identify the functional properties of consciousness, i.e., the fundamental properties related to a functional and causal role on NC processes. I focused on conscious perception and the characteristics of conscious processes, their differences from non-conscious processes, and how the two types of processes interact. I have highlighted that the conscious component of perception allows us to know how objects extend into the space they belong to and overlap with each other and, at a more complex level, what the world around us is like. Secondly, I have put forward a tentative hypothesis of how consciousness might perform this function.

### 6.1 Does the identified property meet the requirements for the function?

1 The identified function is closely related to the properties of experience. The fact that an object extends in a certain way into space is phenomenal evidence. However, the extended nature concerns a content and not a process (Earl, 2014), so one might think that a content extended in a certain way is the conscious result of NC processes. Given the limitations of NC processes, it is plausible that a conscious content does not result only from these processes. Just as it is hard to understand how a qualitative sensation arises from NC processes, it is hard to attribute the property of extending into space to point-like processes like NC ones. Furthermore, this would create the paradox in which a richer knowledge is produced by processes that convey poorer knowledge.

Even if it were possible, such a possibility would not make sense precisely because of its functional connotations. While the hypothesis that the properties of consciousness are produced by NC processes may be plausible for qualia, which accompany corresponding NC processes without providing any functional contribution, it is not tenable when considering EF. We would have to admit that a functional property like extended conscious knowledge is a secondary effect of a function performed through point-like processes. Who or what would benefit from transforming knowledge obtained in symbolic-numerical terms into knowledge by images?

2 The function is useful from an adaptive point of view in the sense that it fills limits of the NC function. What does a NC system do in these situations? How does it immediately know the EF of an image or perform functions that can benefit from knowing the EF, such as perception and sensorimotor skills?

A NC device does not seem concerned with how an object extends into space. It addresses these situations using its existing knowledge of complex structures, such as recognition, as well as its extemporaneous knowledge of simple structural aspects, such as calculating distances. In essence, it handles predictable complex structures and non-predictable simple structures. The ability to handle unpredictable yet simple structures through point-like processes may underlie a NC system’s capacity to detect obstacles, as occurs in blindsight (Ajina and Bridge, 2016).

In theory, nothing prevents a NC device from immediately knowing the EF of an image, but it does not seem equipped for this. For example, calculating the distance of all points in a contour from a central point seems to be overly analytical to provide meaningful information. On the other hand, combining preformed parts (Biederman, 1987) does not seem to be sufficient to cope with the variability of shapes.

However, the question is whether it is really important to know the EF of objects, or whether a NC system can cope with the world without knowing it. The limitations of a blindsight patient seem to be an obvious example. However, in natural minds it is difficult to distinguish the influence of conscious processes from the influence of NC ones. These limitations can also be investigated in artificial intelligence. Due to the impressive increase in the computational power of computers, one might assume that the limits of artificial intelligence vis-à-vis processes that we presume require consciousness are relative and gradually decreasing. Two examples concern perceptual skills evaluated using CAPTCHAs to determine if a user is human (Hasan, 2016; Kumar et al., 2021; Dinh and Hoang, 2023), and the so-called Moravec’s paradox, whereby what is easy for us - i.e. sensorimotor skills—is difficult for computers, and vice versa (Moravec, 1988; Pinker, 1994; Sheikh et al., 2023). Various hypotheses have been proposed regarding the causes of these limitations (McCorduck, 2004; Bursztein et al., 2010; Agrawal, 2010; Hannagan et al., 2012; Rotenberg, 2013; Signorelli, 2018; George et al., 2020; Arora, 2023; Dentella et al., 2024), but they are rarely compared with each other (Deng et al., 2024) or analyzed in relation to consciousness (Moravec, 1988; Piletsky, 2019; Korteling et al., 2021).

CAPTCHAs typically consist of words and objects that are somewhat recognizable but have extreme and unpredictable deviations from how they are usually presented. In the real world, sensorimotor skills are applied through interaction with objects, i.e., complex and unpredictable structures that extend in a certain way into space, by an extended body of complex and variable shape.

Thus, the problem in both cases is the immediate handling of structures extended into space that have complex and unpredictable forms. While complex, the chess world and the symbolic-linguistic world addressed by a Large Language Model can be likened to a world made up of points located in a space. The limits of a NC system consist in immediately coping with a world made of objects extended into space, i.e., the existence in the distal stimulus of complex structures that are neither known nor predictable and refer to the EF of objects in the world.

These limits could be at the origin of the appearance of consciousness in an evolutionary perspective (Lacalli, 2024). Various approaches to consciousness attempt to address the challenge of coping with the complexity and variability of the stimulus, yet they fail to take into account the extended nature of the objects in the world (Earl, 2014; Graziano and Webb, 2015; Seth, 2021).

3 How does consciousness perform a function in a non-computational way? It is not easy to conceive it, to the point that even finding the words to describe it is challenging. Typically, we use the term information processing, which is associated with computation and thus refers to a sequence of actions—whether serial or parallel—performed on point elements. The hypothesis that consciousness is a self-organizing extended reference system is compatible with the possibility of knowing what the world is like through non-computational processes. It seems that conscious knowledge is based on the degree to which an object deviates from a condition of equilibrium in relation to the space to which it belongs. One hypothesis of how this might occur is that the system functions as a “weak” force field that produces knowledge rather than changes in the contents of the field. At the same time, this knowledge influences non-conscious activity. A number of theories hypothesize that consciousness may be determined by the action of electromagnetic fields (McFadden, 2020; Jones and Hunt, 2023), although their role differs from the one proposed here.

### 6.2 Conscious integration and non-conscious integration

The theory presented in this paper has something in common with the Integrated Information Theory (Tononi, 2004, 2008; Tononi and Koch, 2015), which emphasizes the need to integrate information. What are the differences?

Firstly, the integration I have described is due to the fact that the object—and more generally an image—has its own structure. Therefore, the integration I have described differs from that described by Tononi because it is not an exclusively internal fact. At least primarily, it is a property that characterizes consciousness as knowledge of the surrounding world.

Secondly, conscious integration does not occur through relations between points, or between components that can be traced back to points. It is an integration that occurs in extended form and is based primarily on the relationship between object and space. There is no “linear” interdependence between contents or regions of the field. The HSB ensures a hierarchical organization of spatial belongings based on the relationship between contents and the space they belong to. Each content depends primarily on the space to which it belongs. In the example given by Tononi and Koch (2015), honey and moon are related to each other because they both belong to the honeymoon “space,” which in turn belongs to the surrounding space.

Last but not least, it is not a high level of NC integration of information that determines consciousness. On the contrary, it is conscious integration that enables high levels of NC integration, especially in the immediate term. This may justify the correlation found between consciousness and high levels of integration in neuronal structure (Koch et al., 2016), although the causal action is exactly opposite to the one proposed in the Integrated Information Theory. However, a NC network with a high degree of neuronal connectivity is necessary to assimilate the integration enabled by consciousness (Baars, 1997; Dehaene and Naccache, 2001).

With regard to the issue of space, it is worth mentioning the Integrated World Modeling Theory (Safron, 2020, 2022), which combines Integrated Information Theory and Global Neuronal Workspace Theory with the Free Energy Principle and Active Inference framework. According to this theory, modules may be conscious only if they entail integrated models with spatial, temporal, and causal coherence for embodied systems and their relationships to environments in which they are embedded. Without such coherence, there can be no means of situating entities relative to each other with specific properties, and so there would be no means of generating an experienceable world.

Integrated World Modeling Theory suggests that we ought to expect all phenomenal content to involve spatial aspects, potentially requiring multi-level processes of spatialization. The posterior “hot zone” (Boly et al., 2017), represents a source of spatial phenomenology, due to its organization as a hierarchy of 2D grids (Haun and Tononi, 2019). This organization might constitute a prerequisite for Spatial Belongings to be nested within one another.

Another aspect that needs to be considered is the relationship between conscious integration and binding (Revonsuo, 1999). Integration often refers to the multisensory nature of reality. But conscious integration is different from binding. Before sensory information from different modalities or submodalities, this paper concerns the integration of information related to the different components of an object’s shape. Secondly, conscious integration brings together different modalities. But it does so with more complex modalities, in that it relates regions that extend in a certain way. This can only occur through overlapping. Overlapping provides much more information than simply putting different sensory aspects together. We know the red color of the apple in reference to its shape and how it overlaps with it. But an apple is not entirely red, and conscious knowledge allows us to know how red and other colors extend into the space of the apple.

### 6.3 What is consciousness for?

Since it should be evident in the domains associated with consciousness, why is it difficult to identify conscious function? One of the most widely accepted hypotheses associates consciousness with the performance of complex tasks, particularly when these are novel, or require flexibility (Velmans, 2012; Earl, 2014; Mogi, 2024). But the matter is more intricate than commonly assumed. One problem is distinguishing between processes that occur in the absence of consciousness and processes that can be attributed to consciousness (Evans, 2008). An additional problem is that what we observe taking place in the presence of consciousness can be attributed only in part to it.

Consciousness is not merely a NC subsystem that specializes in processing novelty. Moreover, it does not usually work alone. Secondary perception is not such without a role for NC processes. In secondary perception we notice changes. However, this does not mean that we do not know the expected image. Otherwise, we could not know how an object changes. It is the NC processes that determine what is new and what consciousness needs to know. To achieve this, they highlight the novelty while placing the familiar elements in the background. Therefore, knowing how reality deviates from what we know is nothing more than a special case of knowing the world.

If we want to understand what consciousness is for, it must be isolated from the influence of NC processes. The sole task of visual consciousness is to know. In some ways, it is virgin knowledge, as ideally occurs in primary perception. It is a knowing machine. A NC system mainly addresses a known world through recognition processes and pre-existing schemata (Raven and Johnson, 1992; Bargh, 1992; Bargh and Chartrand, 1999; Evans, 2003). Or it addresses a world to be known through learning (Kihlstrom, 1993; Lisman and Sternberg, 2013). It is a fundamentally predictive machine, designed to predict the world and modify its predictive capabilities through learning.

In secondary perception, non-conscious processes and conscious processes are closely intertwined. This is one reason why it is difficult to identify the conscious function and why primary perception remains an essential point of observation. The example reported at the beginning of this paper is perhaps the only way to observe the conscious function in its fundamental essence. This close cooperation likely occurs in more complex domains of perception as well. For example, in this perspective, awareness is a form of knowledge in which not only the objects of the world are known, but also the subject and the relationship between the subject and the world. Presumably, these overlap in the NC images that access consciousness. However, awareness—which I do not address in this paper—is not just that.

At least as far as perception is concerned, mind—as a conscious mind—is not just consciousness. It must be understood with the simultaneous and coordinated functioning of consciousness and of the NC system, of a knowing machine and of a predictive machine. Unlike predictive theories (Hohwy, 2013; Marvan and Havlík, 2021; Seth, 2021), I assign the NC system a predominantly predictive role, while consciousness is involved in knowledge acquisition. When we think we know what consciousness does, we actually know what *conscious mind* does. The conscious mind is the result of the intertwining of conscious and NC processes. Consequently, it is essential to distinguish the influence of conscious processes from that of NC processes.

Another reason why we find it difficult to identify the conscious function is due to its private, first-person nature. As we have seen, knowing what the world is like means knowing how the contents of one or more images related to the world extend into space and overlap each other. However, how the contents extend is something we can perceive but cannot describe or even conceive. Rational thinking enables us to produce knowledge as we commonly conceive it and share it through language. However, it forces us to transform conscious extended knowledge into point-like knowledge. Our symbolic-verbal thinking is probably one of the factors that has made it difficult to know the nature of consciousness.

The theory I have presented has three main limitations. First, it does not explain how qualities such as redness or pain arise from physical activity. The limits of this paper do not allow us to address this issue; however, highlighting the extended nature of conscious contents might offer some insight in this direction (Merleau-Ponty, 1945; Gomez, 2025). Second, the hypothesis about how consciousness fulfills this function and its role in the functioning of the conscious mind remains tentative. Third, I have focused almost exclusively on vision. Therefore, the EIT, as presented here, is, at best, a theory of conscious visual perception.

## Data Availability

The original contributions presented in the study are included in the article/supplementary material, further inquiries can be directed to the corresponding author.

## References

[1] Agrawal K. (2010) To study the phenomenon of the Moravec's paradox ArXiv [Preprint] doi: 10.48550/arXiv.1012.3148

[2] Ajina S. Bridge H. (2016). Blindsight and unconscious vision: what they teach us about the human visual system. Neuroscientist 23, 529–541. doi: 10.1177/1073858416673817, PMID: 27777337 PMC5493986

[3] Amoroso R. Rauscher E. A. (2018). Unity of consciousness experience. Nat. Observ Curr. Phys. Theory. doi: 10.1142/9789814324250_0014

[4] Arakaki M. Dozono C. Frolova H. Hebishima H. Inage S. (2023). Modeling of will and consciousness based on the human language: interpretation of qualia and psychological consciousness. Bio Systems 227-228:104890. doi: 10.1016/j.biosystems.2023.104890, PMID: 37061160

[5] Arnheim R. (1974). Art and visual perception. Berkeley: University of California Press.

[6] Arora A. (2023). Moravec's paradox and the fear of job automation in health care. Lancet 402, 180–181. doi: 10.1016/S0140-6736(23)01129-7, PMID: 37453744

[7] Baars B. (1997). In the theater of consciousness. New York, NY: Oxford University Press.

[8] Baars B. J. Franklin S. (2003). How conscious experience and working memory interact. Trends Cogn. Sci. 7, 166–172. doi: 10.1016/S13646613(03)00056-1 12691765

[9] Banks W. P. (1996). How much work can a quale do? Conscious. Cogn. 5, 368–380. doi: 10.1006/ccog.1996.0023 8906408

[10] Bargh J. A. (1992). The ecology of automaticity: toward establishing the conditions needed to produce automatic processing effects. Am. J. Psychol. 105, 181–199. doi: 10.2307/1423027, PMID: 1621880

[11] Bargh J. A. Chartrand T. L. (1999). The unbearable automaticity of being. Am. Psychol. 54, 462–479. doi: 10.1037/0003-066X.54.7.462, PMID: 40658541

[12] Bayne T. (2010). The unity of consciousness. Oxford, UK: Oxford University Press.

[13] Beshkar M. (2020). The QBIT theory of consciousness. Integr. Psychol. Behav. Sci. 54, 752–770. doi: 10.1007/s12124-020-09528-1, PMID: 32291583

[14] Biederman I. (1987). Recognition-by-components: a theory of human image understanding. Psychol. Rev. 94, 115–147. doi: 10.1037/0033-295X.94.2.115, PMID: 3575582

[15] Billard A. Kragic D. (2019). Trends and challenges in robot manipulation. Science 364:eaat8414. doi: 10.1126/science.aat8414, PMID: 31221831

[16] Blackmore S. (2016). What if consciousness has no function? Behav. Brain Sci. 39:e171. doi: 10.1017/S0140525X15002010, PMID: 28355826

[17] Block N. (1995). On a confusion about a function of consciousness. Behav. Brain Sci. 18, 227–247. doi: 10.1017/S0140525X00038188

[18] Block N. (2011). Perceptual consciousness overflows cognitive access. Trends Cogn. Sci. 15, 567–575. doi: 10.1016/j.tics.2011.11.001, PMID: 22078929

[19] Boly M. Massimini M. Tsuchiya N. Postle B. R. Koch C. Tononi G. (2017). Are the neural correlates of consciousness in the front or in the back of the cerebral cortex? Clinical and neuroimaging evidence. J. Neurosci. 37, 9603–9613. doi: 10.1523/JNEUROSCI.3218-16.2017 28978697 PMC5628406

[20] Brogaard B. Chomański B. Gatzia D. E. (2021). Consciousness and information integration. Synthese 198, 763–792. doi: 10.1007/s11229-020-02613-3

[21] Brown R. Lau H. LeDoux J. E. (2019). Understanding the higher-order approach to consciousness. Trends Cogn. Sci. 23, 754–768. doi: 10.1016/j.tics.2019.06.009, PMID: 31375408

[22] Bursztein E. Bethard S. Fabry C. Mitchell J. Jurafsky D. (2010) How good are humans at solving CAPTCHAs? A large scale evaluation. Proceedings - IEEE Symposium on Security and Privacy.

[23] Cabanac M. Cabanac A. J. Parent A. (2009). The emergence of consciousness in phylogeny. Behav. Brain Res. 198, 267–272. doi: 10.1016/j.bbr.2008.11.028, PMID: 19095011

[24] Calì C. (2020). “Form Constancy” in Glossary of morphology. Lecture Notes in Morphogenesis. eds. Vercellone F. Tedesco S. (Cham: Springer).

[25] Carruthers P. (2004). Phenomenal consciousness: A naturalistic theory. Cambridge: Cambridge University Press.

[26] Chalmers D. J. (1995). Facing up to the problem of consciousness. J. Conscious. Stud. 2, 200–219.

[27] Chalmers D. J. (1996). The conscious mind: In search of a fundamental theory. Oxford: Oxford University Press.

[28] Chella A. Manzotti R. (2016). “The causal roots of integration and the unity of consciousness!” in Biophysics of consciousness: A foundational approach. eds. Poznanski R. Tuszynski J. Feinberg T. (Singapore: World Scientific Press), 189–229.

[29] Crick F. Koch C. (1998). Consciousness and neuroscience. Cereb. Cortex 8, 97–107. doi: 10.1093/cercor/8.2.97, PMID: 9542889

[30] Damasio A. R. (2021). Feeling & knowing: Making minds conscious. New York: Pantheon Books. 10.1080/17588928.2020.1846027 33323038

[31] Damasio A. Carvalho G. B. (2013). The nature of feelings: evolutionary and neurobiological origins. Nat. Rev. Neurosci. 14, 143–152. doi: 10.1038/nrn3403, PMID: 23329161

[32] Damasio A. Meyer K. (2009). “Consciousness: an overview of the phenomenon and of its possible neural basis” in The neurology of consciousness. eds. Laureys S. Tononi G. (London: Academic Press).

[33] Dehaene S. (2014). Consciousness and the brain: Deciphering how the brain codes our thoughts. New York: Viking Penguin.

[34] Dehaene S. Kerszberg M. Changeux J. P. (1998). A neuronal model of a global workspace in effortful cognitive tasks. Proc. Natl. Acad. Sci. USA 95, 14529–14534. doi: 10.1073/pnas.95.24.14529, PMID: 9826734 PMC24407

[35] Dehaene S. Naccache L. (2001). Towards a cognitive neuroscience of consciousness: basic evidence and a workspace framework. Cognition 79, 1–37. doi: 10.1016/S0010-0277(00)00123-2, PMID: 11164022

[36] Deng G. Ou H. Liu Y. Zhang J. Zhang T. Liu Y. (2024). Oedipus: LLM-enchanced reasoning CAPTCHA solver. ArXiv [Preprint]. doi: 10.48550/arXiv.2405.07496

[37] Dentella V. Günther F. Murphy E. Marcus G. Leivada E. (2024). Testing AI on language comprehension tasks reveals insensitivity to underlying meaning. Sci. Rep. 14:28083. doi: 10.1038/s41598-024-79531-8, PMID: 39543236 PMC11564762

[38] Dinh N. T. Hoang V. T. (2023). Recent advances of captcha security analysis: a short literature review. Proc. Comput. Sci. 218, 2550–2562. doi: 10.1016/j.procs.2023.01.229

[39] Earl B. (2014). The biological function of consciousness. Front. Psychol. 5:697. doi: 10.3389/fpsyg.2014.00697, PMID: 25140159 PMC4122207

[40] Edelman G. M. (2003). Naturalizing consciousness: a theoretical framework. Proc. Natl. Acad. Sci. USA 100, 5520–5524. doi: 10.1073/pnas.0931349100, PMID: 12702758 PMC154377

[41] Eikelboom J. A. J. de Knegt H. J. Klaver M. van Langevelde F. van der Wal T. Prins H. H. T. (2020). Inferring an animal's environment through biologging: quantifying the environmental influence on animal movement. Mov. Ecol. 8:40. doi: 10.1186/s40462-020-00228-4, PMID: 33088572 PMC7574229

[42] Evans J. S. B. T. (2003). In two minds: dual-process accounts of reasoning. Trends Cogn. Sci. 7, 454–459. doi: 10.1016/j.tics.2003.08.012, PMID: 14550493

[43] Evans J. S. B. T. (2008). Dual-processing accounts of reasoning, judgment, and social cognition. Annu. Rev. Psychol. 59, 255–278. doi: 10.1146/annurev.psych.59.103006.093629, PMID: 18154502

[44] Feinberg T. E. Mallatt J. (2013). The evolutionary and genetic origins of consciousness in the Cambrian period over 500 million years ago. Front. Psychol. 4:667. doi: 10.3389/fpsyg.2013.00667, PMID: 24109460 PMC3790330

[45] Feldman J. (2013). The neural binding problem(s). Cogn. Neurodyn. 7, 1–11. doi: 10.1007/s11571-012-9219-8, PMID: 24427186 PMC3538094

[46] Forti B. (2009). How could phenomenal consciousness be involved in mental function? New Ideas Psychol. 27, 312–325. doi: 10.1016/j.newideapsych.2008.10.001

[47] Forti B. (2024a). Approaching the nature of consciousness through a phenomenal analysis of early vision. What is the explanandum? Front. Psychol. 15:1329259. doi: 10.3389/fpsyg.2024.1329259, PMID: 38562232 PMC10982490

[48] Forti B. (2024b). The hidden structure of consciousness. Front. Psychol. 15:1344033. doi: 10.3389/fpsyg.2024.1344033, PMID: 38650907 PMC11033517

[49] Frankish K. (2012). Quining diet qualia. Conscious. Cogn. 21, 667–676. doi: 10.1016/j.concog.2011.04.001, PMID: 21543237

[50] Fu Y. Yan W. Shen M. Chen H. (2021). Does consciousness overflow cognitive access? Novel insights from the new phenomenon of attribute amnesia. Sci. China Life Sci. 64, 847–860. doi: 10.1007/s11427-020-1831-8, PMID: 33515433

[51] Fumerton R. (1995). Metaepistemology and skepticism. Lanham, MD: Rowman and Littlefield.

[52] Gallotto S. Sack A. T. Schuhmann T. de Graaf T. A. (2017). Oscillatory correlates of visual consciousness. Front. Psychol. 8:1147. doi: 10.3389/fpsyg.2017.01147, PMID: 28736543 PMC5500655

[53] Garud R. (1997). On the distinction between know-how, know-why, and know-what. Adv. Strat. Manage. 14, 81–101.

[54] George D. Lázaro-Gredilla M. Guntupalli J. S. (2020). From CAPTCHA to commonsense: how brain can teach us about artificial intelligence. Front. Comput. Neurosci. 14:554097. doi: 10.3389/fncom.2020.554097, PMID: 33192426 PMC7645629

[55] Gomez J. D. (2025). The harder problem of consciousness: reflections on a 50-year quest for the alchemy of qualia. Front. Psychol. 16:1592628. doi: 10.3389/fpsyg.2025.1592628 40438765 PMC12116507

[56] Graziano M. S. Webb T. W. (2015). The attention schema theory: a mechanistic account of subjective awareness. Front. Psychol. 6:500. doi: 10.3389/fpsyg.2015.00500, PMID: 25954242 PMC4407481

[57] Grinde B. (2024). Consciousness makes sense in the light of evolution. Neurosci. Biobehav. Rev. 164:105824. doi: 10.1016/j.neubiorev.2024.105824, PMID: 39047928

[58] Grossberg S. (2017). Towards solving the hard problem of consciousness: the varieties of brain resonances and the conscious experiences that they support. Neural Netw. 87, 38–95. doi: 10.1016/j.neunet.2016.11.003, PMID: 28088645

[59] Guberman S. (2017). Gestalt theory rearranged: Back to Wertheimer. Front. Psychol. 8:1782. doi: 10.3389/fpsyg.2017.01782, PMID: 29075220 PMC5641857

[60] Halligan P. W. Oakley D. A. (2021). Giving up on consciousness as the ghost in the machine. Front. Psychol. 12:571460. doi: 10.3389/fpsyg.2021.571460, PMID: 33995166 PMC8121175

[61] Hameroff S. Penrose R. (2014). Consciousness in the universe: a review of the 'Orch OR' theory. Phys Life Rev 11, 39–78. doi: 10.1016/j.plrev.2013.08.002, PMID: 24070914

[62] Hannagan T. Ktori M. Chanceaux M. Grainger J. (2012). Deciphering CAPTCHAs: what a Turing test reveals about human cognition. PLoS One 7:e32121. doi: 10.1371/journal.pone.0032121, PMID: 22396750 PMC3291547

[63] Hasan A. (2016). A survey of current research on CAPTCHA. Int. J. Comput. Sci. Appl. 7:7301. doi: 10.5121/ijcses.2016.7301

[64] Haun A. Tononi G. (2019). Why does space feel the way it does? Towards a principled account of spatial experience. Entropy 21:1160. doi: 10.3390/e21121160

[65] Hayashi Y. Sato R. (2024). The unity of consciousness and the practical ethics of neural organoid research. Neuroethics 18:3. doi: 10.1007/s12152-024-09574-1

[66] Hill C. S. (2018). Unity of consciousness. WIREs Cogn. Sci. 9:e1465. doi: 10.1002/wcs.1465, PMID: 29809308

[67] Hohwy J. (2013). The predictive mind. Oxford, GB: Oxford University Press UK.

[68] Hunt T. Jones M. McFadden J. Delorme A. Hales C. G. Ericson M.. (2024). Editorial: electromagnetic field theories of consciousness: opportunities and obstacles. Front. Hum. Neurosci. 17:1342634. doi: 10.3389/fnhum.2023.1342634, PMID: 38495476 PMC10941648

[69] Husserl E. (1913). Ideas pertaining to a pure phenomenology and to a phenomenological philosophy—First book: General introduction to a pure phenomenology. The Hague: Nijhoff.

[70] Jacquey L. Baldassarre G. Santucci V. G. O'Regan J. K. (2019). Sensorimotor contingencies as a key drive of development: from babies to robots. Front. Neurorobot. 13:98. doi: 10.3389/fnbot.2019.00098, PMID: 31866848 PMC6904889

[71] James W. (1890). The principles of psychology. London: MacMillan.

[72] Jerath R. Braun M. Barnes V. (2015). Functional representation of vision within the mind: a visual consciousness model based in 3D default space. J. Med. Hypoth. Ideas 13:1. doi: 10.1016/j.jmhi.2015.02.001

[73] Jones M. W. Hunt T. (2023). Electromagnetic-field theories of qualia: can they improve upon standard neuroscience? Front. Psychol. 14:1015967. doi: 10.3389/fpsyg.2023.1015967, PMID: 37325753 PMC10267331

[74] Kak S. (2024). “On the non-computability of consciousness” in Consciousness studies in sciences and humanities: Eastern and Western perspectives. eds. Satsangi P. S. Horatschek A. M. Srivastav A. (Cham: Springer Verlag), 77–86.

[75] Kanai R. Chang A. Yu Y. de Magrans Abril I. Biehl M. Guttenberg N. (2019). Information generation as a functional basis of consciousness. Neurosci. Conscious. 2019:niz016. doi: 10.1093/nc/niz016, PMID: 31798969 PMC6884095

[76] Kanai R. Tsuchiya N. (2012). Qualia. Curr. Biol. 22, R392–R396. doi: 10.1016/j.cub.2012.03.033, PMID: 22625852

[77] Kanizsa G. (1979). Organization in vision Essays in gestalt perception. New York: Praeger.

[78] Kanizsa G. (1980). Grammatica del vedere. Bologna: Il Mulino.

[79] Kanizsa G. (1991). Vedere e pensare. Bologna: Il Mulino.

[80] Kauffman S. Roli A. (2022). What is consciousness? Artificial intelligence, real intelligence, quantum mind and qualia. Biol. J. Linn. Soc. 139, 530–538. doi: 10.1093/biolinnean/blac092

[81] Kihlstrom J. F. (1993). “The psychological unconscious and the self” in Experimental and theoretical studies of consciousness, Ciba foundation symposium. eds. Bock G. R. Marsh J. (Chichester: Wiley), 147–167.

[82] Kind A. (2008). “How to believe in qualia” in The case for qualia. ed. Wright E. (Cambridge, MA: MIT Press), 285–298.

[83] Koch C. (2004). The quest for consciousness: A neurobiological approach. Englewood, CO: Roberts & Company.

[84] Koch C. Massimini M. Boly M. Tononi G. (2016). Neural correlates of consciousness: progress and problems. Nat. Rev. Neurosci. 17, 307–321. doi: 10.1038/nrn.2016.22, PMID: 27094080

[85] Köhler W. (1922). Gestaltprobleme und Anfänge einer Gestalttheorie. Jahresber. ges. Physiol. 3, 512–539.

[86] Korteling J. E. van de Boer-Visschedijk G. C. Blankendaal R. A. M. Boonekamp R. C. Eikelboom A. R. (2021). Human- versus artificial intelligence. Front. Artif. Intell. 4:622364. doi: 10.3389/frai.2021.622364 33981990 PMC8108480

[87] Kumar M. Jindal M. K. Kumar M. (2021). A systematic survey on CAPTCHA recognition: types, creation and breaking techniques. Arch. Comput. Methods Eng. 29, 1107–1136. doi: 10.1007/s11831-021-09608-4

[88] Lacalli T. (2024). The function(s) of consciousness: an evolutionary perspective. Front. Psychol. 15:1493423. doi: 10.3389/fpsyg.2024.1493423, PMID: 39660268 PMC11628302

[89] Lamme V. A. (2010). How neuroscience will change our view on consciousness. Cogn. Neurosci. 1, 204–220. doi: 10.1080/17588921003731586, PMID: 24168336

[90] Lamme V. A. F. (2020). Visual functions generating conscious seeing. Front. Psychol. 11:83. doi: 10.3389/fpsyg.2020.00083, PMID: 32116908 PMC7034432

[91] Lisman J. Sternberg E. J. (2013). Habit and nonhabit systems for unconscious and conscious behavior: implications for multitasking. J. Cogn. Neurosci. 25, 273–283. doi: 10.1162/jocn_a_00319, PMID: 23163411

[92] Loorits K. (2014). Structural qualia: a solution to the hard problem of consciousness. Front. Psychol. 5:237. doi: 10.3389/fpsyg.2014.00237, PMID: 24672510 PMC3957492

[93] Luccio R. (2003). The emergence of Prägnanz: Gaetano Kanizsa's legacies. Axiomathes 13, 365–387. doi: 10.1023/B:AXIO.0000007206.84315.ed

[94] Luccio R. (2019). Perceptual simplicity: the true role of prägnanz and Occam. Gestalt Theory 41, 263–276. doi: 10.2478/gth-2019-0024

[95] Ludwig D. (2023). The functions of consciousness in visual processing. Neurosci. Conscious. 2023:18. doi: 10.1093/nc/niac018, PMID: 36628118 PMC9825248

[96] Lycan W. (1996). Consciousness and experience. Cambridge, MA: MIT Press.

[97] Mangan B. (1998). “Against functionalism: consciousness as an information bearing medium” in Toward a science of consciousness II, the second Tucson discussions and debates. eds. Hameroff S. R. Kaszniak A. W. Scott A. C. (Cambridge, MA: MIT Press), 135–142.

[98] Marvan T. Havlík M. (2021). Is predictive processing a theory of perceptual consciousness? New Ideas Psychol. 61:100837. doi: 10.1016/j.newideapsych.2020.100837

[99] Mason J. W. D. (2021). Model Unity and the Unity of consciousness: developments in expected float entropy minimisation. Entropy 23:1444. doi: 10.3390/e23111444, PMID: 34828142 PMC8624278

[100] Masrour F. (2020). “The phenomenal Unity of consciousness” in The Oxford handbook of the philosophy of consciousness. ed. Kriegel U. (Oxford: Oxford University Press), 208–229.

[101] McCorduck P. (2004). Machines who think: A personal inquiry into the history and prospects of artificial intelligence. Natick, MA: A.K. Peters.

[102] McFadden J. (2020). Integrating information in the brain’s EM field: the cemi field theory of consciousness. Neurosci. Conscious. 2020:niaa016. doi: 10.1093/nc/niaa016, PMID: 32995043 PMC7507405

[103] McFadden J. (2023). Consciousness: matter or EMF? Front. Hum. Neurosci. 16:1024934. doi: 10.3389/fnhum.2022.1024934, PMID: 36741784 PMC9889563

[104] McGinn C. (2004). “What constitutes the mind-body problem?” in Consciousness and its objects (Oxford, GB: Oxford University Press).

[105] Merleau-Ponty M. (1945). Phénoménologie de la perception. Paris: Gallimard.

[106] Micher N. Lamy D. (2023). The role of conscious perception in semantic processing: testing the action trigger hypothesis. Conscious. Cogn. 107:103438. doi: 10.1016/j.concog.2022.103438, PMID: 36450219

[107] Mogi K. (2024). Artificial intelligence, human cognition, and conscious supremacy. Front. Psychol. 15:1364714. doi: 10.3389/fpsyg.2024.1364714 38807956 PMC11130558

[108] Moraresku S. Vlcek K. (2020). The use of egocentric and allocentric reference frames in static and dynamic conditions in humans. Physiol. Res. 69, 787–801. doi: 10.33549/physiolres.934528, PMID: 32901499 PMC8549915

[109] Moravec H. P. (1988). Mind children: The future of robot and human intelligence. Cambridge, MA: Harvard University Press.

[110] Morsella E. (2005). The function of phenomenal states: supramodular interaction theory. Psychol. Rev. 112, 1000–1021. doi: 10.1037/0033-295X.112.4.1000, PMID: 16262477

[111] Murgia M. Prpic V. Santoro I. Sors F. Agostini T. Galmonte A. (2016). Perceptual belongingness determines the direction of lightness induction depending on grouping stability and intentionality. Vis. Res. 126, 69–79. doi: 10.1016/j.visres.2015.10.018, PMID: 27208582

[112] Nagel T. (1974). What is it like to be a bat? Philos. Rev. 83, 435–450. doi: 10.2307/2183914

[113] Nichols S. Grantham T. (2000). Adaptive complexity and phenomenal consciousness. Philos. Sci. 67, 648–670. doi: 10.1086/392859

[114] Niikawa T. Miyahara K. Hamada H. Nishida S. (2022). Functions of consciousness: conceptual clarification. Neurosci. Conscious. 2022:6. doi: 10.1093/nc/niac006, PMID: 35356269 PMC8963277

[115] Noel J. P. Ishizawa Y. Patel S. R. Eskandar E. N. Wallace M. T. (2019). Leveraging nonhuman primate multisensory neurons and circuits in assessing consciousness theory. J. Neurosci. 39, 7485–7500. doi: 10.1523/JNEUROSCI.0934-19.2019, PMID: 31358654 PMC6750944

[116] Orpwood R. (2017). Information and the origin of qualia. Front. Syst. Neurosci. 11:22. doi: 10.3389/fnsys.2017.00022, PMID: 28484376 PMC5399078

[117] Overgaard M. (2018). Phenomenal consciousness and cognitive access. Philos. Trans. R. Soc. B 373:20170353. doi: 10.1098/rstb.2017.0353, PMID: 30061466 PMC6074085

[118] Piletsky E. (2019). Consciousness and unconsciousness of artificial intelligence. Future Human Image 11, 66–71. doi: 10.29202/fhi/11/7, PMID: 40549439

[119] Pinker S. (1994). The language instinct: How the mind creates language. New York, NY: HarperCollins.

[120] Pockett S. (2004). Does consciousness cause behaviour? J. Conscious. Stud. 11, 23–40.

[121] Raven P. H. Johnson G. B. (1992). Biology. 3rd Edn. St. Louis, MO: Mosby Year.

[122] Reeves A. Dresp-Langley B. (2017). Perceptual categories derived from Reid’s “common sense” philosophy. Front. Psychol. 8:893. doi: 10.3389/fpsyg.2017.00893, PMID: 28634457 PMC5459909

[123] Reid T. (1764/1977) in An inquiry into the human mind on the principles of common sense. ed. Brookes D. R. (Edinburgh: Edinburgh University Press).

[124] Revonsuo A. (1999). Binding and the phenomenal unity of consciousness. Conscious. Cogn. 8, 173–185. doi: 10.1006/ccog.1999.0384, PMID: 10448000

[125] Robinson W. S. (2007). Evolution and epiphenomenalism. J. Conscious. Stud. 14, 27–42.

[126] Rosenthal D. (1997). “A theory of consciousness” in The nature of consciousness: Philosophical debates. eds. Block N. Flanagan O. Güzeldere G. (Cambridge, MA: MIT Press/Bradford Books), 729–753.

[127] Rosenthal D. (2008). Consciousness and its function. Neuropsychologia 46, 829–840. doi: 10.1016/j.neuropsychologia.2007.11.012, PMID: 18164042

[128] Rotenberg V. S. (2013). Moravec’s paradox: consideration in the context of two brain hemisphere functions. Act. Nerv. Super. 55, 108–111. doi: 10.1007/BF03379600

[129] Russel B. (1912). The problems of philosophy. London: Williams and Norgate.

[131] Safron A. (2020). An integrated world modeling theory (IWMT) of consciousness: combining integrated information and global neuronal workspace theories with the free energy principle and active inference framework; toward solving the hard problem and characterizing agentic causation. Front. Artif. Intell. 3:30. doi: 10.3389/frai.2020.00030, PMID: 33733149 PMC7861340

[132] Safron A. (2022). Integrated world modeling theory expanded: implications for the future of consciousness. Front. Comput. Neurosci. 16:642397. doi: 10.3389/fncom.2022.642397, PMID: 36507308 PMC9730424

[133] Samaha J. (2015). How best to study the function of consciousness? Front. Psychol. 6:604. doi: 10.3389/fpsyg.2015.00604, PMID: 25999907 PMC4423300

[134] Searle J. (1992). The rediscovery of mind. Cambridge, MA: MIT Press.

[135] Searle J. (2004). Mind. A brief introduction. New York: Oxford University Press.

[136] Seth A. K. (2021). Being you: a new science of consciousness. New York: Dutton, an imprint of Penguin Random House LLC.

[137] Seth A. K. Bayne T. (2022). Theories of consciousness. Nat. Rev. Neurosci. 23, 439–452. doi: 10.1038/s41583-022-00587-4, PMID: 35505255

[138] Sheikh H. Prins C. Schrijvers E. (2023). “Artificial intelligence: definition and background” in The new system technology. ed. Mission A. I. (Cham: Springer).

[139] Shepard R. N. Metzler J. (1971). Mental rotation of three-dimensional objects. Science 171, 701–703. doi: 10.1126/science.171.3972.701, PMID: 5540314

[140] Shoemaker S. (1991). Qualia and consciousness. Mind C, 507–524. doi: 10.1093/mind/C.400.507, PMID: 40084282

[141] Signorelli C. M. (2018). Can computers become conscious and overcome humans? Front. Robot. AI 5:121. doi: 10.3389/frobt.2018.00121, PMID: 33501000 PMC7805878

[142] Solms M. (2021). The hidden spring: A journey to the source of consciousness. New York, NY: W.W. Norton & Company.

[143] Song D. (2007). Non-computability of consciousness. ArXiv [Preprint] doi: 10.48550/arXiv.0705.1617

[144] Strupp W. (2024). A new variant of the electromagnetic field theory of consciousness: approaches to empirical confirmation. Front. Neurol. 15:1420676. doi: 10.3389/fneur.2024.1420676, PMID: 39494171 PMC11527664

[145] Sturm T. (2012). Consciousness regained? Philosophical arguments for and against reductive physicalism. Dialogues Clin. Neurosci. 14, 55–63. doi: 10.31887/DCNS.2012.14.1/tsturm, PMID: 22577305 PMC3341650

[146] Tononi G. (2004). An information integration theory of consciousness. BMC Neurosci. 5:42. doi: 10.1186/1471-2202-5-42, PMID: 15522121 PMC543470

[147] Tononi G. (2008). Consciousness as integrated information: a provisional manifesto. Biol. Bull. 215, 216–242. doi: 10.2307/25470707, PMID: 19098144

[148] Tononi G. Edelman G. M. (1998). Consciousness and complexity. Science 282, 1846–1851. doi: 10.1126/science.282.5395.1846, PMID: 9836628

[149] Tononi G. Koch C. (2008). The neural correlates of consciousness: an update. Ann. N. Y. Acad. Sci. 1124, 239–261. doi: 10.1196/annals.1440.004, PMID: 18400934

[150] Tononi G. Koch C. (2015). Consciousness: here, there and everywhere? Philos. Trans. R. Soc. Lond. Ser. B Biol. Sci. 370, 1–18. doi: 10.1098/rstb.2014.0167 PMC4387509 25823865

[151] Tsytsarev V. (2022). Methodological aspects of studying the mechanisms of consciousness. Behav. Brain Res. 419:113684. doi: 10.1016/j.bbr.2021.113684, PMID: 34838578

[152] Tuszynski J. A. (2020). From quantum chemistry to quantum biology: a path toward consciousness. J. Integr. Neurosci. 19, 687–700. doi: 10.31083/j.jin.2020.04.393, PMID: 33378843

[153] Tyler C. W. (2020). Ten testable properties of consciousness. Front. Psychol. 11:1144. doi: 10.3389/fpsyg.2020.01144, PMID: 32670141 PMC7326790

[154] Umiltá C. (2007). “Consciousness and control of action” in The Cambridge handbook of consciousness. eds. Zelazo P. D. Moscovitch M. Thompson E. (Cambridge: Cambridge University Press), 327–351.

[155] Vallortigara G. (2021). The rose and the fly. A conjecture on the origin of consciousness. Biochem. Biophys. Res. Commun. 564, 170–174. doi: 10.1016/j.bbrc.2020.11.005, PMID: 33213842

[156] Van Geert E. Wagemans J. (2023). Prägnanz in visual perception. Psychon. Bull. Rev. 31, 541–567. doi: 10.3758/s13423-023-02344-9, PMID: 37787874 PMC11061049

[157] Velmans M. (1991). Is human information processing conscious? Behav. Brain Sci. 14, 651–726. doi: 10.1017/S0140525X00071776

[158] Velmans M. (2002). How could conscious experiences affect brains? J. Conscious. Stud. 9, 3–29.

[159] Velmans M. (2012). The evolution of consciousness. Contemp. Soc. Sci. 7, 117–138. doi: 10.1080/21582041.2012.692099

[160] Wagemans J. Feldman J. Gepshtein S. Kimchi R. Pomerantz J. R. van der Helm P. A.. (2012). A century of gestalt psychology in visual perception: I conceptual and theoretical foundations. Psychol Bull. 138, 1218–1252. doi: 10.1037/a0029334, PMID: 22845750 PMC3728284

[161] Ward L. Guevara R. (2022). Qualia and phenomenal consciousness arise from the information structure of an electromagnetic field in the brain. Front. Hum. Neurosci. 16:874241. doi: 10.3389/fnhum.2022.874241, PMID: 35860400 PMC9289677

[162] Wegner D. M. (2002). The illusion of conscious will. Cambridge, MA: MIT Press.

[163] Wertheimer M. (1923). Untersuchungen zur Lehre von der Gestalt, II. Psychol. Forsch. 4, 301–350. doi: 10.1007/BF00410640

[164] Wiese W. (2022). Attentional structure and phenomenal unity. Open Philos. 5, 254–264. doi: 10.1515/opphil-2022-0197

[165] Wiest M. (2025). A quantum microtubule substrate of consciousness is experimentally supported and solves the binding and epiphenomenalism problems. Neurosci. Conscious. 2025:11. doi: 10.1093/nc/niaf011, PMID: 40342554 PMC12060853

[166] Wikipedia Contributors (2025). Theory of forms. In Wikipedia, the free encyclopedia. Available online at https://en.wikipedia.org/w/index.php?title=Theory_of_forms&oldid=1284410399 (Accessed April 26, 2025).

[167] Witzel C. Gegenfurtner K. R. (2018). Color perception: objects, Constancy, and categories. Annu. Rev. Vis. Sci. 4, 475–499. doi: 10.1146/annurev-vision-091517-034231, PMID: 30004833

[168] Xue J. Li C. Quan C. Lu Y. Yue J. Zhang C. (2017). Uncovering the cognitive processes underlying mental rotation: an eye-movement study. Sci. Rep. 7:10076. doi: 10.1038/s41598-017-10683-6, PMID: 28855724 PMC5577169

[169] Yurchenko S. B. (2022). From the origins to the stream of consciousness and its neural correlates. Front. Integr. Neurosci. 16:928978. doi: 10.3389/fnint.2022.928978, PMID: 36407293 PMC9672924

[170] Zeman A. (2001). Consciousness. Brain 124, 1263–1289. doi: 10.1093/brain/124.7.1263, PMID: 11408323

[171] Zhi G. Xiu R. (2023). Quantum theory of consciousness. J. Appl. Math. Phys. 11, 2652–2670. doi: 10.4236/jamp.2023.119174

